# Introduction to *Bilingualism and Cognitive Control*

**DOI:** 10.3389/fpsyg.2013.00199

**Published:** 2013-04-17

**Authors:** I. K. Christoffels, J. F. Kroll, M. T. Bajo

**Affiliations:** ^1^Department of Psychology, Faculty of Psychology and Leiden Institute for Brain and Cognition, University of LeidenLeiden, Netherlands; ^2^Department of Psychology, Pennsylvania State University, University ParkPA, USA; ^3^Departamento de Psicología Experimental, Universidad de GranadaGranada, Spain

In the past decade, there has been an upsurge of research on bilingualism. A theme in this work is that the bilingual's two languages are always active, at times converging with one another to produce benefits to comprehension and production, but at other times conflicting, with the requirement to negotiate cross-language competition. A goal in the recent work has been to characterize the cognitive processes that enable bilinguals to negotiate the cross-talk between their two languages. The ease with which highly proficient bilinguals are able to speak each of their languages without frequent errors or intrusions and, at the same time, switch between the two languages in contexts in which code switching is appropriate or encouraged, suggests the presence of a high level of cognitive control. At the same time, behavioral and neurocognitive studies have shown that bilinguals differ from monolinguals in their performance on tasks that are purely cognitive, often showing advantages relative to monolinguals, and clear differences in neural function and structure. A key question is how we might begin to relate the findings on language control to the documented cognitive consequences of bilingualism. The papers in this special issue on *Bilingualism and Cognitive Control* represent the best of the new research on each of these issues to understand how control in language processing is achieved and how domain-general cognitive processes are themselves affected by language experience.

In what follows, we review and summarize the main goals of the papers that comprise this effort. We note that the questions about cognitive control are increasingly exploiting sophisticated methods (e.g., see the work using delta plot analysis, Roelofs et al., 2011) and extending analyses of executive function to different populations of language users (e.g., Tao et al., 2011). We set out in this special topic issue to ask a number of questions concerning cognitive control in bilingualism. The contributing authors addressed these questions in very different and interesting ways.

A still controversial issue is: *How is cognitive control manifest during bilingual language processing?* Calabria et al. (2012) asked whether bilingual language control is the same as other types of cognitive control. The pattern of symmetrical switch costs that is often obtained for proficient bilinguals did not replicate across domain in a non-linguistic switch task in the same group. Therefore, the authors argue that bilingual language control is not completely subsidiary to domain general control. Their conclusions contrast to those of Roelofs et al. (2011) who used delta-plot analyses to show that telltale characteristics of the reaction time distribution in bilingual naming performance shows clear similarities with performance in other domains. This finding supports the view that inhibition is a mechanism of attentional control in bilingual language performance, and that it is a domain general mechanism. In their review of patient studies, intracranial electrical, and transcranial magnetic stimulation, Hervais-Adelman et al. (2011) proposed a distinction between two distinct networks contributing to the executive control of language. A fronto-basal-ganglia loop is implicated in the inhibition of the irrelevant language during production, and may be crucial for access to translation equivalents. A fronto-parietal network appears to subserve more general switching mechanisms. Perhaps such a distinction may be helpful in resolving controversies that have arisen in behavioral studies of switching and control.

van Heuven et al. (2011) used the Stroop task to address the effects of cross-language similarity. Three groups of trilingual's systematically differed on whether their languages use the same or different scripts. They obtained similar within-language Stroop interference across groups, but between-language Stroop interference was modulated by cross-language similarity, in particular by differences in script between languages. Hoshino and Thierry (2012) used Event Related Potentials (ERP) to investigate interlingual homographs. These stimuli induce between language semantic conflict because they have identical form, but different meanings in both languages. Both readings of interlingual homographs were processed, even in a single language context. Interlingual homographs modulated the N400 time window during word reading. Interestingly, there was no effect in later time windows suggesting that the activated semantic information of the non-target language is not explicitly processed. Adaptive performance during learning was addressed by Davidson and Indefrey (2011) who also used ERPs to investigate how learning of grammatical categories leads to changing error-related electrophysiological activity over time. They showed that learning only took place when performance feedback was provided. Finally, Morales et al. (2011) report a study in which the manifestation of cognitive control is investigated in the hitherto unexplored direction of grammatical gender. Their study suggested the presence of an inhibitory mechanism that suppresses grammatical gender when it is a source of competition between languages.

The idea that bilingualism may result in cognitive advantages is a topic that has received a great deal of attention in the recent scientific and popular literature: *What aspects of cognitive control are enhanced for proficient bilinguals?* Recent evidence suggests that (only) specific aspects of executive control are related to bilingualism. Papers in the special issue support this claim by demonstrating specific cognitive advantages associated with bilingualism. Tao et al. (2011) found bilingual enhancement of executive functions for early and late bilinguals. They investigated how age of L2 acquisition and relative balance of the two languages influenced performance on a lateralized attention network test (ANT) for executive function. Monolinguals were less efficient in the resolution of conflict than both early and late bilinguals. Although both early and late bilinguals were found to have more efficient attentional networks, late bilinguals showed the greatest advantage in conflict resolution, whereas early bilinguals were advantaged in monitoring. By testing simultaneous interpreters, Yudes et al. (2011) explored non-verbal executive processes in a bilingual group known to have exceptional working memory abilities. They compared interpreters, bilinguals and monolinguals on the Wisconsin Card Sorting Task and the Simon task, taken to reflect cognitive flexibility and inhibitory control, respectively. Interpreters showed higher mental flexibility than the other groups. However, a similar Simon effect indicated that interpreters do not out perform other groups on inhibitory control of executive functioning. Investigating a rather different aspect of cognitive functioning, Hommel et al. (2011) addressed the relation between bilingualism and creativity. They showed a specific advantage for high proficient participants for convergent thinking. In contrast, low proficient bilinguals were better at a divergent thinking task. This suggests that bilingualism supports a relative focused cognitive control style, with strong top-down control.

A number of papers in the special issue addressed language switching habits as an index of individual differences rather than focusing on the more general patterns of switch costs themselves. Festman and Münte (2012) used performance on a switch task to identify participants as switchers or non-switchers based on the degree of unintentional switching during naming. They found that non-switchers were advantaged on aspects of the Wisconsin Card Sorting Task and the Flanker task, indicating that individual differences in language control and executive control function are related. Rodriguez-Fornells et al. (2012) developed a questionnaire to psychometrically assess self-perceived individual differences in language switching. Soveri et al. (2011) used this questionnaire combined with a multiple regression approach to investigate whether performance on tasks measuring different executive functions could be predicted by the frequency of language switches in everyday life.

A related focus on individual differences in another set of papers concerned the question of *How individual differences in cognitive resources influence second language learning and performance*. Bartolotti et al. (2011) asked whether cognitive control and bilingual experiences influence success in learning a new language. They tested groups of participants with high and low cognitive control abilities and high and low bilingual experience. Results indicated that both factors may influence learning success; their relative importance depends of the amount of overlap between languages. In the high interference condition cognitive control abilities influenced learning success. Pivneva et al. (2012) addressed fluency and nativeness in the L1 and L2 spontaneous monologue and dialogue. Not only proficiency levels influenced speech planning and production, but these processes were also more efficient for bilinguals with high inhibitory capacity, in particular for highly proficient bilinguals. Finally, Reiterer et al. (2011) examined EEG gamma band phase synchrony measures for high and low proficient participants. Processing in the second language required significantly higher synchronization strength than in the first language. Lower proficiency was related to a stronger synchronized network than higher proficiency, which was more widely distributed in left fronto-parietal areas.

As should be clear, this special topic has generated many exiting new contributions to some of the most important questions that relate to bilingualism and cognitive control. An issue that has not been addressed in this set of papers, is the question how language environment affects concurrent processing. We anticipate that the context of language use and language learning will become a topic of interest and investigation in the next wave of research on bilingualism and control. That said, the current set of papers offers a fresh and novel perspective on how cognitive control is engaged to enable proficient language performance and how skilled bilingual performance changes cognition in ways that suggest much greater optimism about the plasticity of the adult mind and brain than previously understood.
